# Large language models facilitating modern molecular biology and novel drug development

**DOI:** 10.3389/fphar.2024.1458739

**Published:** 2024-12-24

**Authors:** Xiao-huan Liu, Zhen-hua Lu, Tao Wang, Fei Liu

**Affiliations:** ^1^ School of Biological Science, Jining Medical University, Jining, China; ^2^ College of Chemical and Biological Engineering, Zhejiang University, Hangzhou, China

**Keywords:** artificial intelligence, large language models, drug development, ChatGPT, protein structure prediction

## Abstract

The latest breakthroughs in information technology and biotechnology have catalyzed a revolutionary shift within the modern healthcare landscape, with notable impacts from artificial intelligence (AI) and deep learning (DL). Particularly noteworthy is the adept application of large language models (LLMs), which enable seamless and efficient communication between scientific researchers and AI systems. These models capitalize on neural network (NN) architectures that demonstrate proficiency in natural language processing, thereby enhancing interactions. This comprehensive review outlines the cutting-edge advancements in the application of LLMs within the pharmaceutical industry, particularly in drug development. It offers a detailed exploration of the core mechanisms that drive these models and zeroes in on the practical applications of several models that show great promise in this domain. Additionally, this review delves into the pivotal technical and ethical challenges that arise with the practical implementation of LLMs. There is an expectation that LLMs will assume a more pivotal role in the development of innovative drugs and will ultimately contribute to the accelerated development of revolutionary pharmaceuticals.

## 1 Introduction

During the past few decades, the field of drug discovery has undergone a transformative revolution, largely due to the rapid advancements in information technology and modern biotechnology, such as artificial intelligence (AI), machine learning (ML), structural revolution (crystallography), and synthetic biology (Sadybekov and Katritch, 2023; Murray et al., 2023; Cova et al., 2022; Roggia et al., 2024). A notable paradigm shift is evident in contemporary drug discovery, where emerging technologies have streamlined the drug development process, consequently reducing associated costs (Pandey et al., 2022). Among them, computational approaches guided by molecular modeling techniques with AI for “hit identification” and “lead optimization” have garnered significant interest from biotech firms and research institutions (Jayatunga et al., 2022; Chakraborty et al., 2023). “Hit identification” is the process of screening large compound libraries to discover molecules that exhibit initial biological activity against a specific target, serving as potential starting points for drug development. “Lead optimization” is the systematic process of refining and enhancing the potency, selectivity, and pharmacokinetic properties of a promising drug candidate to improve its therapeutic potential and reduce side effects. Nowadays, the strategic application of AI in drug discovery has significantly hastened the development timeline and diminished both the cost and duration of early-stage drug discovery phases (Chakraborty et al., 2023; Lamberti et al., 2019).

LLMs are cutting-edge AI systems crafted on the foundation of neural network architectures and refined through exposure to human language from a plethora of sources, including articles, books, and news reports. Consequently, LLMs are capable of capture the complicated associative relationships between words in a text-based training dataset, harnessing the capabilities of deep learning (Thirunavukarasu et al., 2023). These models have been effectively integrated into numerous fields, demonstrating a versatility that encompasses dialogue and beyond. In particular, the recent surge in advancements within LLMs have paved the way for their integration into healthcare and biotechnological pharmaceutics (Liu et al., 2023a). Boasting the capacity to execute a multitude of language-centric tasks, LLMs capitalize on neural networks and are trained on vast repositories of text generated by humans, thus transforming them into invaluable assets for information retrieval and the delivery of biomedical insights. Consequently, they can serve as valuable tools for retrieving information and providing biomedical solutions (Thirunavukarasu et al., 2023; Eggmann et al., 2023). Among the vanguard of AI-powered LLMs, ChatGPT stands as a notable example, which was instrumental in streamlining the drug discovery process (De Angelis et al., 2023). Typically, LLMs in drug development could be utilized for understanding disease mechanisms, designing and optimizing drug molecules, predicting efficacy and safety, integrating with AI tools, translating between molecules and indications, and exploring federated learning for enhanced data utilization and task generalization. Especially, LLMs can be integrated with other AI technologies like machine learning and computational biology tools to synergistically accelerate drug discovery. For example, machine learning algorithms can analyze vast databases to identify intricate patterns, leading to the discovery of novel therapeutic targets and prediction of potential drug candidates with better accuracy and speed. Quantitative structure-activity relationship (QSAR) modeling and molecular docking simulations are AI-driven predictive techniques that provide insights into predicting the biological activity of novel compounds with great accuracy.

There is a strong belief that the rapid evolution and widespread adoption of AI are defining trends of our time. In this review, the recent development of LLMs was highlighted, including their architectural frameworks and operational mechanisms. Furthermore, the manuscript has placed a special emphasis on the practical applications of Large Language Models (LLMs) in the biopharmaceutical sector. Several successful case studies were detailed to highlight the strengths and limitations of these models. Each case study includes an in-depth analysis and critical evaluation of the respective models. It is especially crucial to diligently evaluate and address the associated concerns, risks, and potential pitfalls (Borji, 2023). It is believed that the integration of LLMs in drug discovery would greatly facilitate the acceleration of the drug discovery pipeline.

## 2 Designing an artificial intelligence-driven platform

### 2.1 Large language models (LLMs) for drug development

Armed with the capabilities of natural language processing (NLP) and machine learning technologies, chatbots have demonstrated significant potential and have made substantial contributions across several fields (Haque and Rubya, 2023; Xu et al., 2021; Suppadungsuk et al., 2023). In particular, the emergence of ChatGPT, with its harnessing of generative models, has heightened global awareness of the vast of generative AI (Sallam, 2023). As this technology continues to evolve, this section aims to offer a comprehensive of the recent advances in LLMs within the realm of biotechnological pharmaceutics.

#### 2.1.1 ChatGPT

The chat generative pretrained transformer (ChatGPT), developed by OpenAI, stands at the forefront of language model-based chatbots, renowned for its conversational interactivity (https://openai.com/blog/chatgpt). On 14 March 2023, an upgraded version, GPT-4, was launched, which boasts improved capabilities for addressing complex issues with heightened precision and rationality. Furthering its progression, on 6 November 2023, the state-of-the-art model, GPT-4 Turbo, was introduced. This iteration is marked by its superior performance, an updated knowledge cutoff date of April 2023, and the introduction of a 128k context window, equating to the processing capacity of approximately 300 pages of text within a single prompt. Utilizing a neural network to process natural language, it is adept at generating contextually relevant responses and delivering nuanced, sophisticated answers through advanced modeling techniques (Brown et al., 2020).

Generally, ChatGPT can be harnessed in the following capacities. Primarily, ChatGPT serves as an intuitive interface a that facilitates more straightforward interactions between users and various AI systems, offering an alternative to traditional knowledge graph navigation. Indeed, it has emerged as a leading example of sophisticated human–computer interaction (HCI). Secondly, ChatGPT can be specifically applied for drug discovery, functioning as an advanced search engine tailored to the nuances of biological science in different ways (Savage, 2023). For rational drug design, ChatGPT could be used to generate innovative chemical structures with a high potential for clinical success and predict the absorption, distribution, metabolism, excretion, and toxicity (ADMET) profiles of the identified compounds (Savage, 2023; Zhao and Wu, 2023). Within this domain, efficient screening can be performed with ultra-large virtual libraries (greatly expanded drug-like chemical spaces), which would significantly amplify the drug-like chemical spaces and enhancing the probability of hit identification and lead discovery. Thirdly, ChatGPT holds promise in the generation of new protein targets for drug development. When equipped with extensive unlabeled data (e.g., the nearly 250 million protein sequences contained in the UniProt database and 1.28 million protein sequences contained in the PDB database), ChatGPT can autonomously deduce the intricate relationships between molecular building blocks on its own. In this field, its functionality mirrors that of AlphaFold, which depends heavily on (new) big data analytics and artificial intelligence for its operations.

**FIGURE 1:**
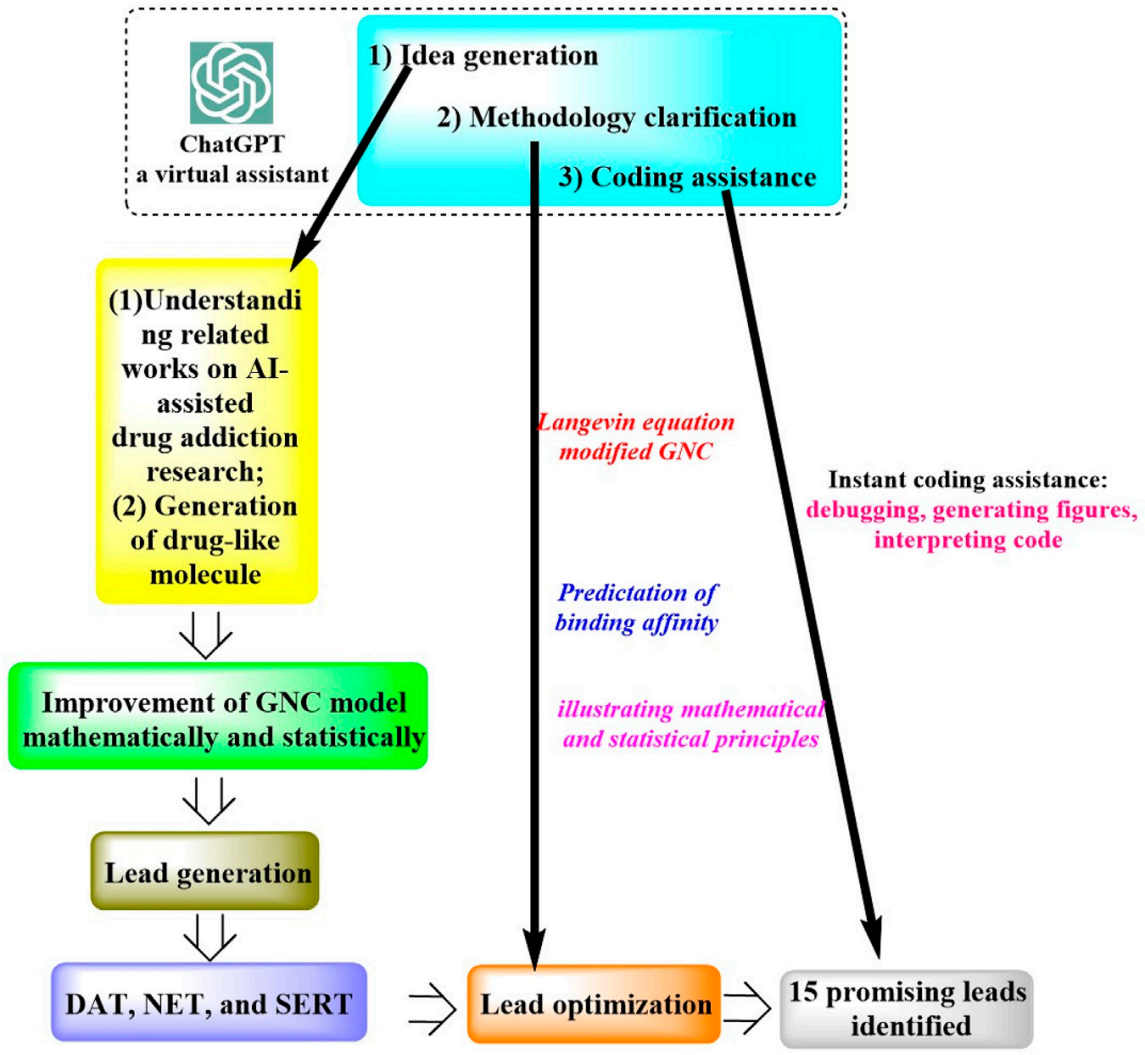
Schematic representation of the ChatGPT-assisted drug addiction research process. (This process initiates with grasping the roles of AI in drug addiction research and generating drug-like molecules. It advances by refining the GNC model to produce leads targeting DAT, NET, and SERT receptors. Optimization is driven by the Langevin equation and an enhanced GNC model, prioritizing binding affinity predictions, which led to 15 potential drug leads pinpointed. ChatGPT supports this process by offering creative input, methodological insights, and coding aid, from debugging to interpretation).

**FIGURE 2:**
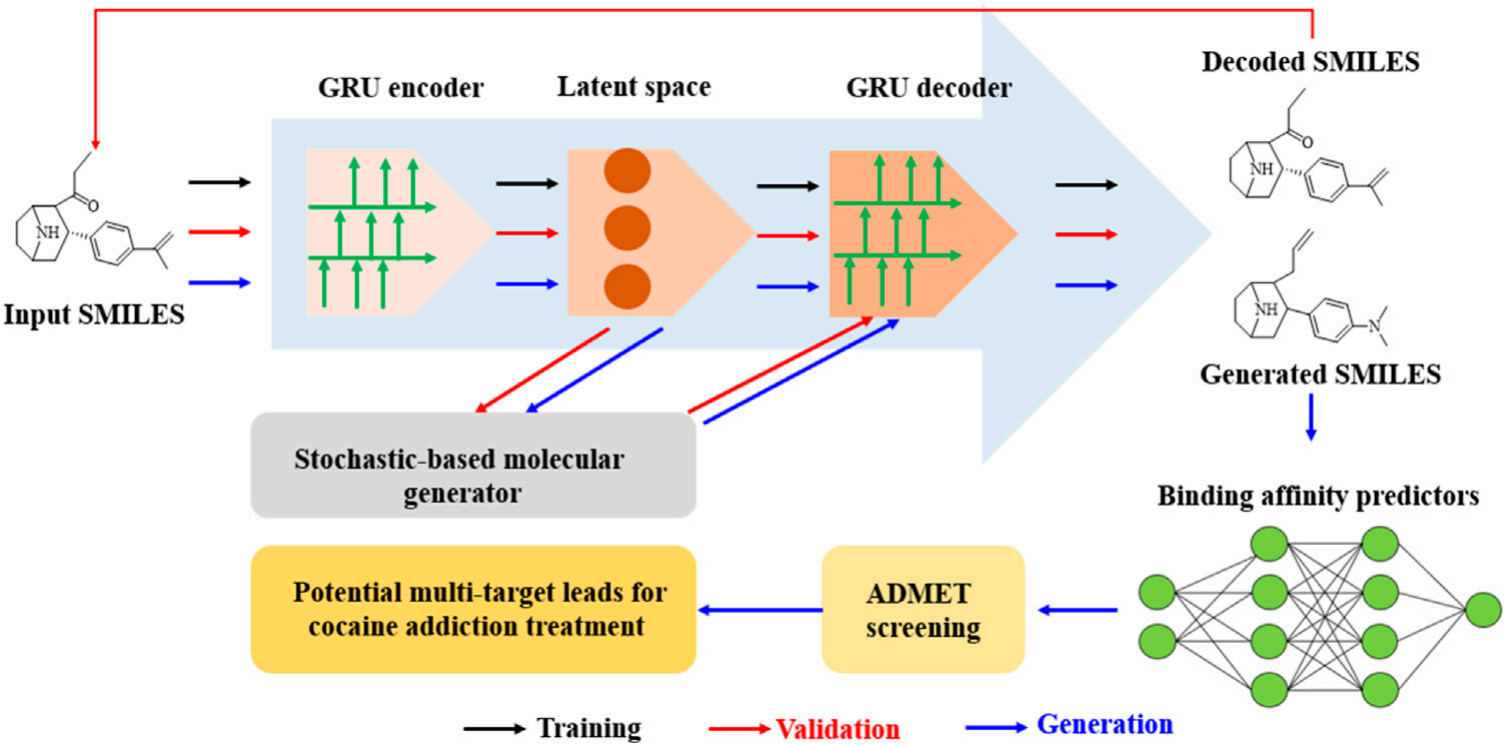
Schematic of a GRU-enhanced autoencoder for drug design: from SMILES encoding to cocaine addiction treatment leads. (The molecular generation pipeline uses a GRU-based autoencoder to encode and decode SMILES strings, and the stochastic generator was used to create novel molecules. The process starts from input SMILES through latent space manipulation to generated SMILES. Subsequently ADMET screening and binding affinity prediction were achieved for the identification of potential cocaine addiction treatment leads).

In a study by Wang et al. (2023a), ChatGPT was successfully applied for the discovery of an anti-cocaine-addiction drug, which functions as a virtual guide offering strategic and methodological insights, as well as generative models for optimal drug-like molecules with desired properties (Figure 1). With the aid of ChatGPT, a novel platform named the Stochastic Generative Network Complex (SGNC) was developed. With this project, ChatGPT primarily serves for idea generation, methodology clarification, and coding assistance (Figure 2). For idea generation, ChatGPT was augmented with three plugins (WebPilot, ScholarAI, and AskYourPDF), which improves its capacity to comprehend the research background of anti-cocaine-addiction drug development, providing up-to-date available sources and accessing insights from previous works. For methodology clarification, plugins (WebPilot, Link Reader and Wolfram) were used to significantly improve the mathematical and statistical capabilities of ChatGPT. In terms of coding assistance, WebPilot, ChatwithGit, and Prompt Perfect were leveraged to refine coding skills and craft perfect prompts.

ChatGPT has been instrumental in successful identification of 15 promising drug leads capable of targeting the dopamine transporter (DAT), norepinephrine transporter (NET), and serotonin transporter (SERT). It was clearly indicated that the “cognitive abilities” of ChatGPT have the potentials to significantly streamline the development of modern pharmaceuticals offering potential promising avenues for drug discovery. However, it was also noted that the application of ChatGPT for drug development still faces many challenges due to the inherent limitations of generative AI. For example, it is still susceptible to generate false narratives and spread misinformation. Consequently, it is recommended that the information generated by ChatGPT-4 undergo rigorous and consistent verification to ensure its accuracy and reliability.

**FIGURE 3:**
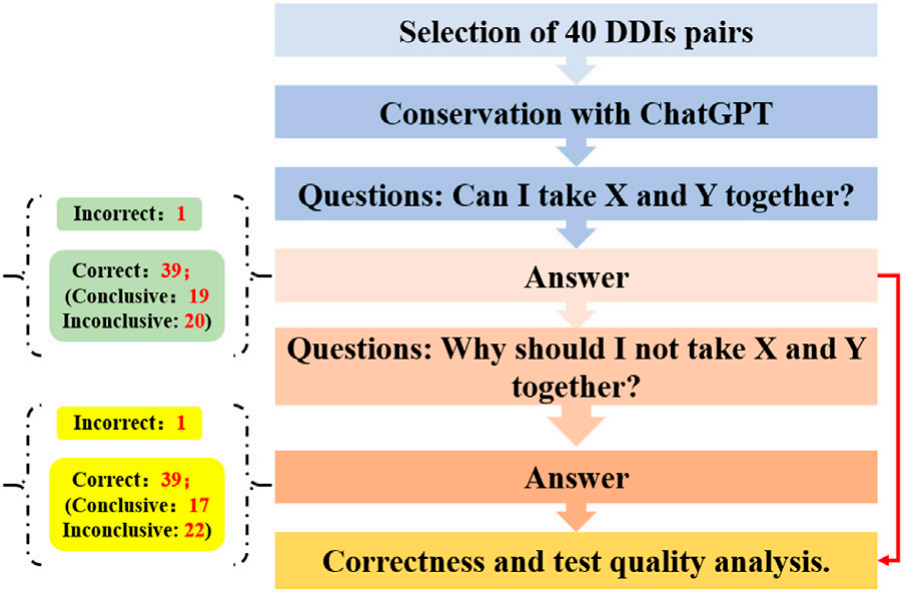
The study flowchart to predict and explain drug-drug interactions with ChatGPT.

Mondal et al. explored the proficiency of ChatGPT in predicting and elucidating common drug-drug interactions (DDIs) (Figure 3) (Juhi et al., 2023). Initially, a curated set of 40 pairs of previously listed DDIs were selected for analysis *via* ChatGPT through a two-tiered questioning approach. The outcomes showed that for the initial query, one response was incorrect, while of the correct responses, 19 were definitive and 20 remained ambiguous. For the second question, one answer was deemed incorrect, with 17 correct answers being definitive and 22 being inconclusive. These results suggest that ChatGPT serves as a moderately effective instrument for assessing DDIs; however, it occasionally falls short in offering comprehensive guidance, indicating the necessity for further refinements to enhance its accuracy and reliability.

Zhang et al. investigated the competencies of ChatGPT in the realms of question-answering, knowledge discovery, and knowledge reasoning within the biomedical field, specifically its ability to establish connections between pairs of proposed entities. The performance of ChatGPT was then compared with existing biomedical knowledge graphs (BKGs) (Hou et al., 2023). The findings indicated that ChatGPT-4.0 outperformed BKGs in terms of providing existing knowledge, although BKGs had a higher confidence level and demonstrated higher reliability in terms of information accuracy. Moreover, compared with BKGs, ChatGPT demonstrated a limited ability to perform novel discoveries based on the existing information and to provide reasoning for knowledge discovery. Therefore, the study proposed that strategies integrating LLMs (like ChatGPT) and BKGs could be promising to enhance task performance and mitigate potential risks.

Xu et al. delved into the prowess and promise of ChatGPT within the realm of biomedical information retrieval, with a particular focus on its ability to discern associations between drugs and diseases (Gao et al., 2023). Their findings showed that ChatGPT achieved an impressive accuracy range of 74.6%–83.5% in identifying drug-disease associations and an even more remarkable 96.2%–97.6% for true and false pairs under varying prompt designs. This revealed that ChatGPT could serve as a valuable “assistant” in unearthing knowledge related to biotechnological and pharmaceutical advancements, with a level of reliability that is quite satisfactory. Nevertheless, it was also emphasized that the insights gleaned from ChatGPT should undergo thorough verification before being integrated into clinical practice. In a separate study, ChatGPT was employed to meticulously annotate single-cell RNA sequencing data, successfully correlating rare cell types with their functions and uncovering several distinct differentiation pathways of cell subtypes that had previously eluded detection (Zehua and Du, 2023).

Blatz et al. demonstrated the transformative potential of ChatGPT and other LLMs in the field of dental medicine (Eggmann et al., 2023). It was concluded that LLMs (e.g., ChatGPT) could be instrumental in several areas (Sadybekov and Katritch, 2023): revolutionizing dental practice by streamlining administrative tasks (Murray et al., 2023); enhancing dental telemedicine through real-time language translation services, thereby making consultations more accessible and scalable, particularly in underserved regions (Cova et al., 2022); bolstering clinical decision support by swiftly summarizing voluminous medical records or aggregating evidence-based medical findings (Roggia et al., 2024); expediting administrative tasks such as routine correspondence and record-keeping (Pandey et al., 2022); enriching patient education with credible health advice and guidance (Jayatunga et al., 2022); advancing dental education through the creation and administration of multiple-choice exams, practical assessments, and supervised patient treatments; and (Chakraborty et al., 2023) refining scientific writing, making it more coherent for non-native English speakers. In addition, the study underscored the imperative to address several critical challenges effectively (Sadybekov and Katritch, 2023): ensuring robust cybersecurity measures to safeguard patient data and medical information against malware attacks (Murray et al., 2023); implementing stringent patient data privacy protections to maintain confidentiality and security; and (Cova et al., 2022) conducting thorough scientific evaluations and verifications of LLM-generated responses to maintain accuracy and reliability.

As highlighted in numerous studies, ChatGPT is poised to exert profound influences on various aspects of natural science and social science in the near future. On the one hand, it is imperative to recognize that at present, ChatGPT might not be a fully-fledged “sage” capable of providing responses replete with adequate reasoning and evidence-based justifications (Heck, 2023). Consequently, robust quality control protocols must be established to safeguard the accuracy, credibility, privacy, and cybersecurity of the swift and beneficial information dispensed by ChatGPT. On the other hand, there remains a critical need to improve the “intelligence” of ChatGPT, thereby transforming it into an indispensable asset for researchers.

#### 2.1.2 Google bard and microsoft bing

Bard AI, a cutting-edge large language model developed by Google, signifies a new frontier in the field of AI-powered chatbots (Pichai, 2023). Propelled by the Language Model for Dialogue Applications (LaMDA, a state-of-the-art transformer-based neural language model), Bard is honed on an expansive dataset, ensuring its proficiency in conversational AI. Sharing a repertoire of capabilities with ChatGPT, Google Bard has demonstrated its efficacy across a spectrum of scientific applications, marking it as a formidable contender in the landscape of advanced AI technologies.

Sulaiman et al. conducted a study to assess proficiency of Google Bard in critically evaluating DDI screening, and it was subsequently compared with the authorized Lexicomp^®^ Online™ database (Sulaiman et al., 2023). The interrater reliability analysis revealed a minimal concordance between Lexicomp and Google Bard in assessing DDI risk, with a Cohen’s kappa (κ) value of 0.01; similarly, a slight agreement between was observed in their severity ratings (κ = 0.02). However, there was a lack of consensus regarding the reliability rate, reflected by a κ value of −0.02. In a parallel study, AI platforms (ChatGPT, Bard, and Bing) were applied for DDI the prediction, and the sensitivity, specificity, and accuracy of each model were subsequently evaluated (Al-Ashwal et al., 2023). Notably, Microsoft Bing emerged as the top performer in terms of specificity (0.769) and accuracy (0.788). Furthermore, ChatGPT-3.5 and ChatGPT-4 exhibited the greatest variability in the consistency of their accuracy, highlighting the nuances in their predictive capabilities.

Cheungpasitporn et al. conducted a comparative analysis of the performance of various AI models (ChatGPT 3.5, ChatGPT 4, Bard AI, and Bing Chat) in identifying potassium and phosphorus content in foods (Qarajeh et al., 2023). The study revealed that ChatGPT 4 outperformed others in determining potassium content, achieving an overall accuracy of 81%, with specific rates of 60% for low-potassium foods and an impressive 99% for high-potassium foods. Comparatively, ChatGPT 3.5, Bard AI and Bing Chat showed accuracies of 66%, 79% and 81% accuracy, respectively. In the realm of phosphorus content identification, Bard AI stood out with a perfect 100% accuracy rate; in contrast, ChatGPT 3.5, ChatGPT 4 and Bing Chat managed to correctly identify high-phosphorus foods only 85%, 77% and 89% of the time, respectively. These findings illustrate the promising potential of AI-powered models in supporting renal diet management, particularly as adjunct tools for enhancing nutritional education and counseling. Nonetheless, it is evident that further enhancements are essential to achieve the desired levels of precision and reliability.

Tham et al. evaluated the proficiency of ChatGPT-3.5, ChatGPT-4.0, and Google Bard in generating accurate responses to inquiries concerning myopia (Beutel et al., 2023). Frequently asked myopia care-related questions were categorized into six different domains and allocated to the AI models. The responses generated were subsequently reviewed independently by three expert ophthalmologists, who graded them as poor, borderline, or good. A consensus approach was then used to establish the final assessment of each reply. The results showed that ChatGPT-4.0 outperformed in terms of accuracy, with 80.6% of the responses deemed ‘good’, surpassing 54.8% for Google Bard (Google Bard: 4.35 and ChatGPT-4.0: 4.23). All the models showed high average comprehensiveness scores and significant self-correction capabilities (66.7% for ChatGPT-4.0% and 60% for Google Bard). This highlighted the potential of ChatGPT-4.0 and Google Bard to deliver essential answers to myopia-related queries, although it is clear that their accuracy requires further enhancement and rigorous evaluation.

In a recent investigation, the capacity of ChatGPT and Google Bard to generate professional-quality responses to inquiries regarding ocular symptoms were systematically examined (Pushpanathan et al., 2023). The answers procured were meticulously appraised and graded by ophthalmologists at the consultant level, based on criteria of accuracy, comprehensiveness, and self-awareness. ChatGPT-4.0 achieved an impressive ‘good’ rating of 89.2%, significantly outperforming Google Bard, which registered at 40.5%. Although all the models garnered high mean comprehensiveness scores, they were concurrently found to display inadequate self-awareness capabilities. Parallel results were also observed in the accuracy of ChatGPT and Google Bard when addressing clinical radiology challenges on the Japan Radiology Board Examination (JRBE) (Toyama et al., 2023).

Therefore, although ChatGPT-4.0 demonstrated a distinct advantage in providing logical answers to a broad spectrum of inquiries, rigorous validation remains essential to ensure reliability and accuracy. In the context of research writing and data collection, it is worth noting that while Bard represents a modest improvement over ChatGPT (ChatGPT3.5) in analyzing the diversity of manuscript bibliographies, it still falls short of reference identification capabilities, even with its integration with Google search (King, 2023).

#### 2.1.3 Med-PaLM

**FIGURE 4:**
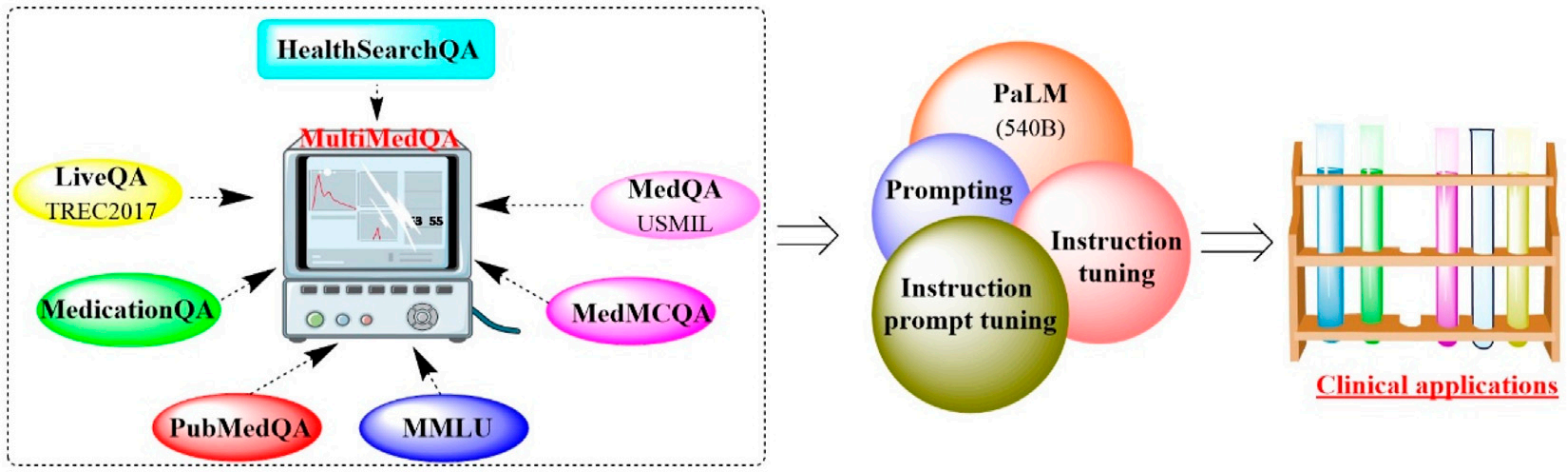
T Overview of the benchmark MultiMedQA, PaLM and Med-PaLM. (Various medical question-answering datasets are integrated into a multimodal framework, which would improve the performance of PaLM through prompting and instruction tuning for clinical applications).

Recently, Google and DeepMind introduced MultiMedQA, which is a comprehensive collection of seven medical question-answering datasets (Figure 4), including LiveQA, MedQA, MedMCQA, MedicationQA, PubMedQA, HealthSearchQA, and MMLU clinical topics (Singhal et al., 2023). Utilizing MultiMedQA as a foundation, both the pathway language model (PaLM) (Chowdhery et al., 2022) and its instruction-tuned derivative, Flan-PaLM, were subjected to rigorous examination (Chung et al., 2022). The results showed that Flan-PaLM excelled in achieving the highest historical accuracy on the aforementioned datasets. Notably, Flan-PaLM scored an impressive 67.6% accuracy on MedQA (US Medical Licensing Exam-style questions), surpassing the previous state of the art by over 17%. These results prompted the implementation of prompt tuning to further specialize Flan-PaLM for the medical domain, resulting in the Med-PaLM model. Med-PaLM was capable of providing more favorable answers to the medical queries than clinicians, although its overall performance was still somewhat inferior to that of medical professionals. This demonstrated the effectiveness of instruction prompt tuning in improving the performance of Med-PaLM.

However, the study also identified limitations and proposed future research direction. These include expanding MultiMedQA to better mirror real-world clinical workflows, developing key LLM capabilities for numerous clinically significant applications, refining human evaluation, and addressing issues of fairness, equity, and ethical considerations.

#### 2.1.4 DrugChat

In a cutting-edge study, a pharmaceutical domain-specific LLM prototype, DrugChat, was developed to utilize ChatGPT-like capabilities for the analysis of drug compounds and the provision of insights on drug–molecule graphs (Liang et al., 2023). Like ChatGPT, DrugChat engages in multi-turn, interactive dialogues to address inquiries about uploaded compound molecule graphs. It is composed of three core components: a graph neural network (GNN), a large language model (LLM), and an adaptor, all of which are trained in an end-to-end fashion.

The GNN is tasked with interpreting the input compound molecule graph and extracting a meaningful representation. The adaptor then converts this graph representation into a format that is compatible with LLM. The LLM processes the transformed compound representation alongside the questions posed about the compound, ultimately generating answers.

For the instruction tuning phase, datasets comprising 10,834 drug compounds and 143,517 question-answer pairs were curated to train DrugChat. In this process, a pretrained GNN and a pre-trained Vicuna-13b model were utilized, with the weight parameters for the GNN and LLMs being fixed. However, the weight parameters for the adapter were continuously refined. The results demonstrated the proficiency of DrugChat in responding to various questions about the input compounds, such as “What makes this compound unique?” and “What diseases might this compound be able to treat?”, even when evaluated on the drug compound graphs not present in the training data.

However, as highlighted, the most significant challenge for DrugChat could be the phenomenon of “artificial (molecular) hallucinations” stemming from the implanted LLMs. The generation of unreliable answers and descriptions could seriously impede its practical application in drug discovery, potentially leading to undesirable consequences.

#### 2.1.5 MolReGPT

**FIGURE 5:**
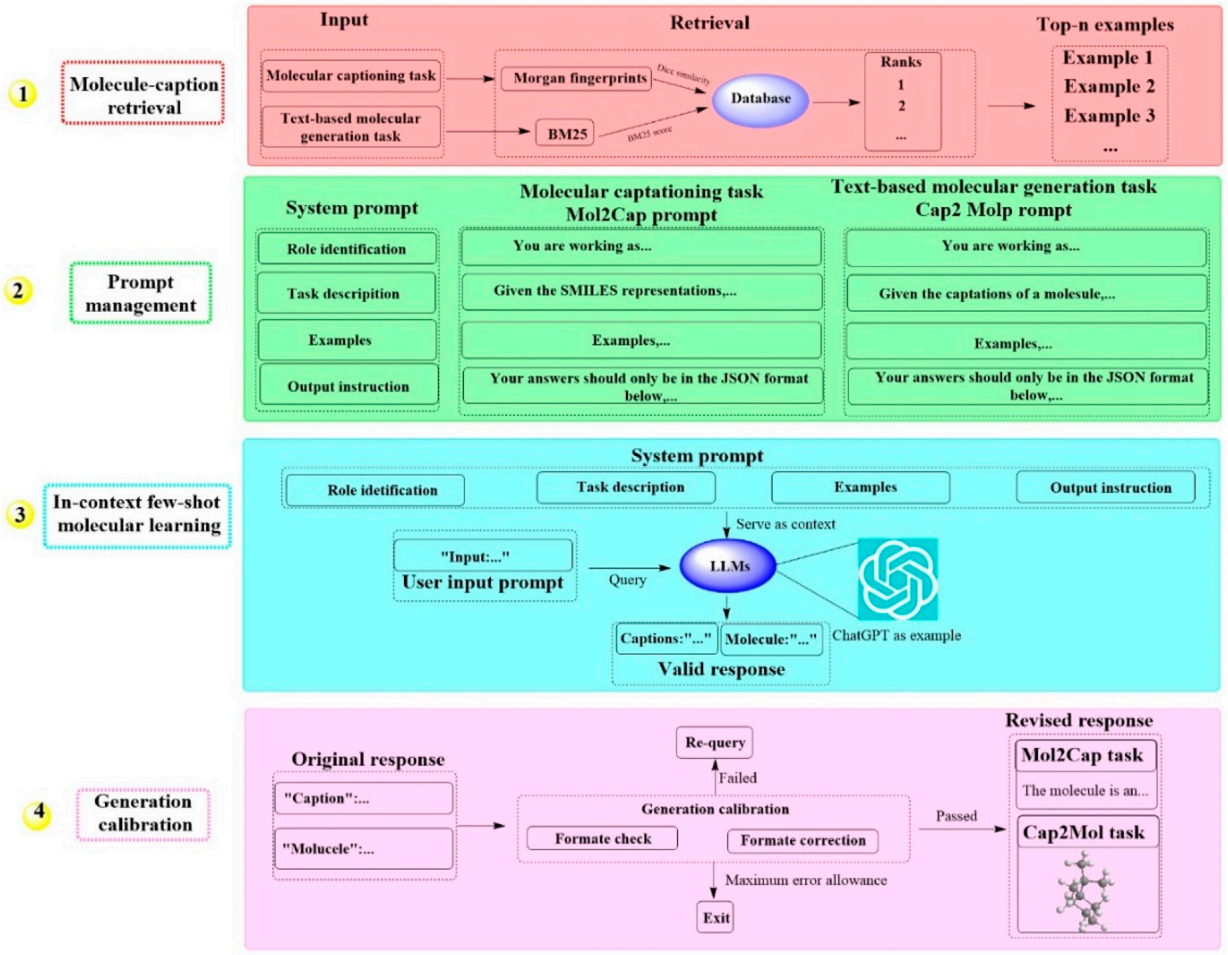
The workflow of MolReGPT. (This figure illustrates a four-step process for generating molecular captions and text-based molecular designs. Initially, it retrieves examples from a database using molecular information. Subsequently, it curates prompts with system instructions for tasks like molecular captioning and generation. In the third phase, it uses a few examples to facilitate a language model learn and generate responses. Finally, it calibrates the generation to ensure the responses are accurate and in the correct format for tasks like Mol2Cap, where it describes molecules).

Recently, a groundbreaking LLM-based system known as MolReGPT has been developed, which showcases the ability to translating molecule captions into natural language (Li et al., 2023). This system employs a retrieval-based prompt paradigm through in-context learning for both molecule captioning and text-based molecule generation. This could potentially revolutionize molecule discovery with MolReGPT without the need for fine-tuning. MolReGPT is structured around four principal components (Figure 5), including molecule caption retrieval (identifying the *n* most analogous examples), prompt management (constructing the system prompt), in-context few-shot molecule learning (translating molecule caption), and generation calibration (assessing validity). For the task of molecule caption retrieval, MolReGPT leverages Morgan fingerprints for molecule captioning and BM25 for text-based molecule generation. Prompt management encompasses four key steps: role identification, task description, example generation, and instruction output.

The results indicated that MolReGPT surpasses the performance of the tested fine-tuned models (e.g., MolT5-base) without any additional fine-tuning, achieving Text2Mol scores of 0.560 for molecule captioning and 0.571 for molecule generation. In terms of molecule understanding and text-based molecule generation, MolReGPT is comparable to the fine-tuned model MolT5-large. These findings suggest that MolReGPT could provide an innovative and adaptable platform for harnessing the potential of LLMs to advance molecule discovery through in-context learning, which might greatly reduce the cost associated with domain transfer.

#### 2.1.6 Chemformer

To tackle the resource-intensive challenge and multitasking demands in cheminformatics, a transformer-based model named Chemformer has been introduced, leveraging SMILES notation (Irwin et al., 2022). Chemformer, which is based on the BART language model, is versatile and can be readily applied to diverse tasks, such as sequence-to-sequence (e.g., reaction prediction and molecular optimization) and discriminative cheminformatics (e.g., property prediction) tasks, with the encoder stack alone being sufficient for many of these applications.

**FIGURE 6:**
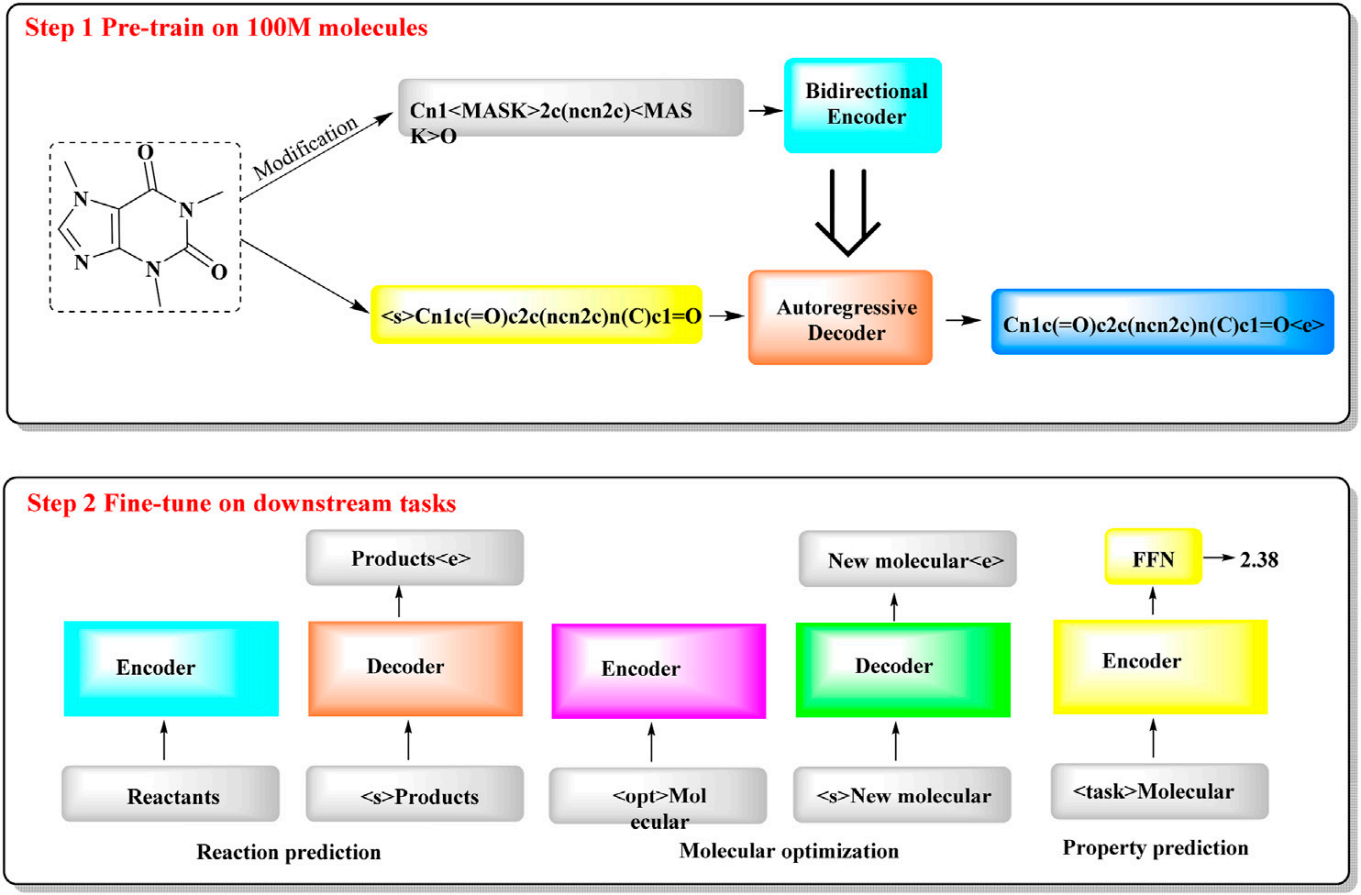
The workflow of Chemformer. (It shows a two-step process for training a molecular model. In Step 1, the model is pre-trained on 100 million molecules, where it learns to encode and decode molecular structures. In Step 2, the model is fine-tuned for specific tasks, such as predicting reactions, optimizing molecules, and estimating molecular properties. Each task uses an encoder to understand the molecule and a decoder to generate the result).

The training of Chemformer primarily consists of two stages (Figure 6): self-supervised pretraining and downstream fine-tuning. In the pretraining phase, extensive unlabeled SMILES datasets are used for model training through three different self-supervised pretraining tasks (masking, augmentation and a combination of masking and augmentation). During the fine-tuning phase, the pretrained Chemformer is tailored to a specific downstream task and further refined. A multitask learning strategy is utilized in this process to optimize multiple tasks concurrently, such as multiproperty prediction and multigene activity prediction. In particular, Chemformer has achieved the highest accuracy available on benchmark datasets for direct synthesis and retrosynthesis prediction.

The outcomes for chemical reaction prediction, molecular optimization and property prediction demonstrate the adaptability of Chemformer to various downstream tasks. The convergence rate and performance of Chemformer on downstream tasks could be improved by self-supervised pretraining. When training time is limited, the synergy of transfer learning and the innovative augmentation strategy can produce state-of-the-art results across all the tested downstream Seq2seq tasks, including chemical reaction prediction and molecular optimization.

#### 2.1.7 MolGPT

**FIGURE 7:**
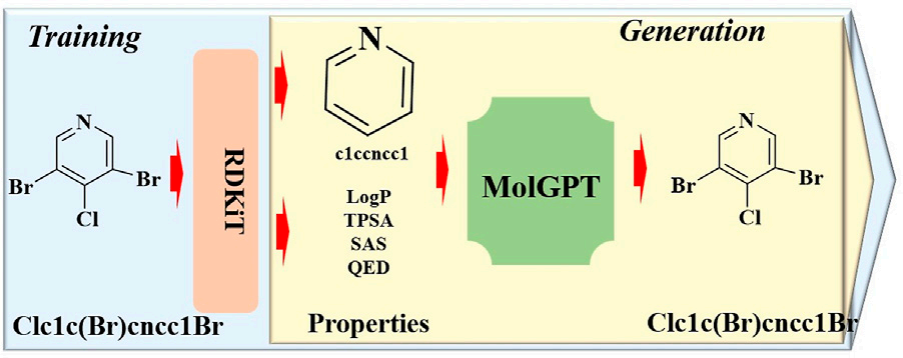
The architectural structure of MolGPT.

To develop a generative pretraining (GPT) model adept at generating chemical structures with tailored properties or synthesizing drug-like molecules, a transformer-decoder-based generative model named MolGPT has been proposed (Bagal et al., 2022). MolGPT is comprised of three principal components (Figure 7): the input encoder, transformer-decoder model, and output decoder. The input encoder translates target molecules represented in the Simplified Molecular Input Line Entry System (SMILES) notation into a string of characters. The transformer-decoder model is comprised of multiple transformer modules and a single decoder module. Each transformer module features a multi-head masked self-attention mechanism calculated by the “Scaled Dot Product Attention”, and a feed-forward network designed to capture contextual information of the input sequence. The decoder module employs both self-attention and an encoder-decoder attention mechanism to generate the subsequent SMILES token. The output decoder then translates the generated SMILES string into a molecular structure. In this process, the resultant molecules can be generated based on desired single or multiple properties, a specified scaffold, or a combination of both.

Benchmarking experiments for training and evaluation demonstrated that MolGPT boasts exceptionally high validity and uniqueness scores, along with commendable Frechet ChemNet Distance (FCD) and KL divergence scores for the MOSES and GuacaMol datasets. Moreover, in terms of validity and novelty, MolGPT outperformed all the other tested methods for the GuacaMol dataset. In addition, MolGPT was found to acquire higher-level chemical representations through molecular property control, enabling the generation of molecules with intriguing properties or specified scaffolds. Based on these results, MolGPT is poised to play a critical role in the realm of rational drug design.

In a recent advancement, a conditional generative pretrained transformer model, cMolGPT, was designed for the autoregressive generation of target-specific *de novo* molecules using natural language processing (NLP) techniques (Wang et al., 2023b). This model was initially pretrained on an extensive SMILES dataset, enabling it to learn the parametric probabilistic distribution of drug-like properties (e.g., LogP and molecular weight), across the SMILES vocabulary space in an unsupervised manner. The cMolGPT model incorporates a of key-value pairs within the transformer architecture, which is further enforced by target-specific embeddings to facilitate the conditional generation of multihead attention for drug-like compounds. The findings revealed that cMolGPT is adept at generating SMILES strings that represent both drug-like and active compounds. In addition, the generated compounds not only closely resemble the chemical space of actual target-specific molecules but also encompass a significant portion of novel compounds. To assess the performance in generating target-specific compounds, evaluations were conducted on three target-biased datasets: EGFR, HTR1A, and S1PR1. The compounds generated by cMolGPT were predicted to exhibit higher activity compared to those generated by the tested conditional RNN models. This suggests that cMolGPT is a promising tool in the field of drug discovery, capable of expanding the chemical space of potential therapeutic agents.

To encapsulate the findings, it is evident that a transformer-decoder-based generative model could achieve state-of-the-art performance in the rational design and discovery of desired chemical structures. Consequently, such a model is regarded as an invaluable asset in the realm of *de novo* drug design, showcasing its potential to revolutionize the way we approach the development of novel therapeutics.

#### 2.1.8 MOLGEN

**FIGURE 8:**
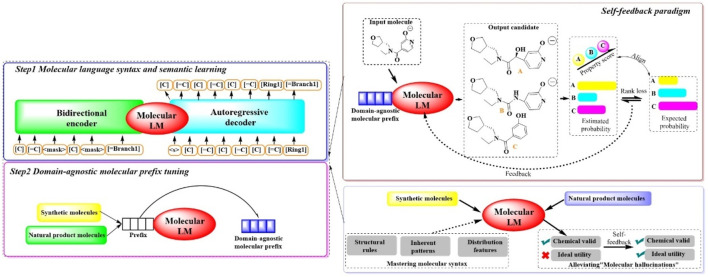
The architectural structure of MOLGEN. (In Step 1, the model learns molecular language syntax and meaning by encoding and decoding molecular structures. In Step 2, the model is fine-tuned with domain-agnostic molecular prefixes to improve the capability of understanding molecular structures. In this process, a self-feedback paradigm was included, where the model is capable of generating candidates, evaluating them based on properties, and learns from the feedback to enhance the performance for generating synthetic and natural product molecules).

To synthesize molecules with specific desired attributes, MOLGEN, a sophisticated pretrained molecular language model, has been recently introduced (Fang et al., 2023b). This system encompasses two pivotal stages (Figure 8): (Sadybekov and Katritch, 2023) a two-stage domain-agnostic molecular pretraining and (Murray et al., 2023) a self-feedback mechanism designed to mitigate the occurrence of “molecular hallucinations”. During the initial phase, the system reconstructs over 100 million molecules using SELFIES, a highly robust molecular language. This approach is complemented by the introduction of the domain-agnostic molecular prefix, which improves the transferability of the knowledge across diverse domains. The subsequent stage introduces a self-feedback paradigm, which is instrumental in fine-tuning the model’s parameters in accordance with generative probabilities, thereby incrementally refining the optimization of the generated molecules. This mechanism is pivotal in enabling MOLGEN to produce molecules with desired properties while circumventing the pitfalls of “molecular hallucinations”.

The efficacy of MOLGEN was subjected to a rigorous evaluation through extensive testing on established benchmarks. The assessments focused on its ability to accurately capture molecular distributions, generate diverse and realistic molecules, pinpoint targeted molecules and refine molecules under constraints. Across the domains of natural products and synthetic molecules, MOLGEN has consistently its proficiency in generating molecules that align with desired chemical preferences (e.g., logP (octanol–water partition coefficient), QED (quantitative estimate of drug likeness). Moreover, it has demonstrated a notable potential for identifying essential molecular substructures and navigating the chemical space, highlighting its value in the realm of molecular design and drug discovery.

#### 2.1.9 KV-PLM

**FIGURE 9:**
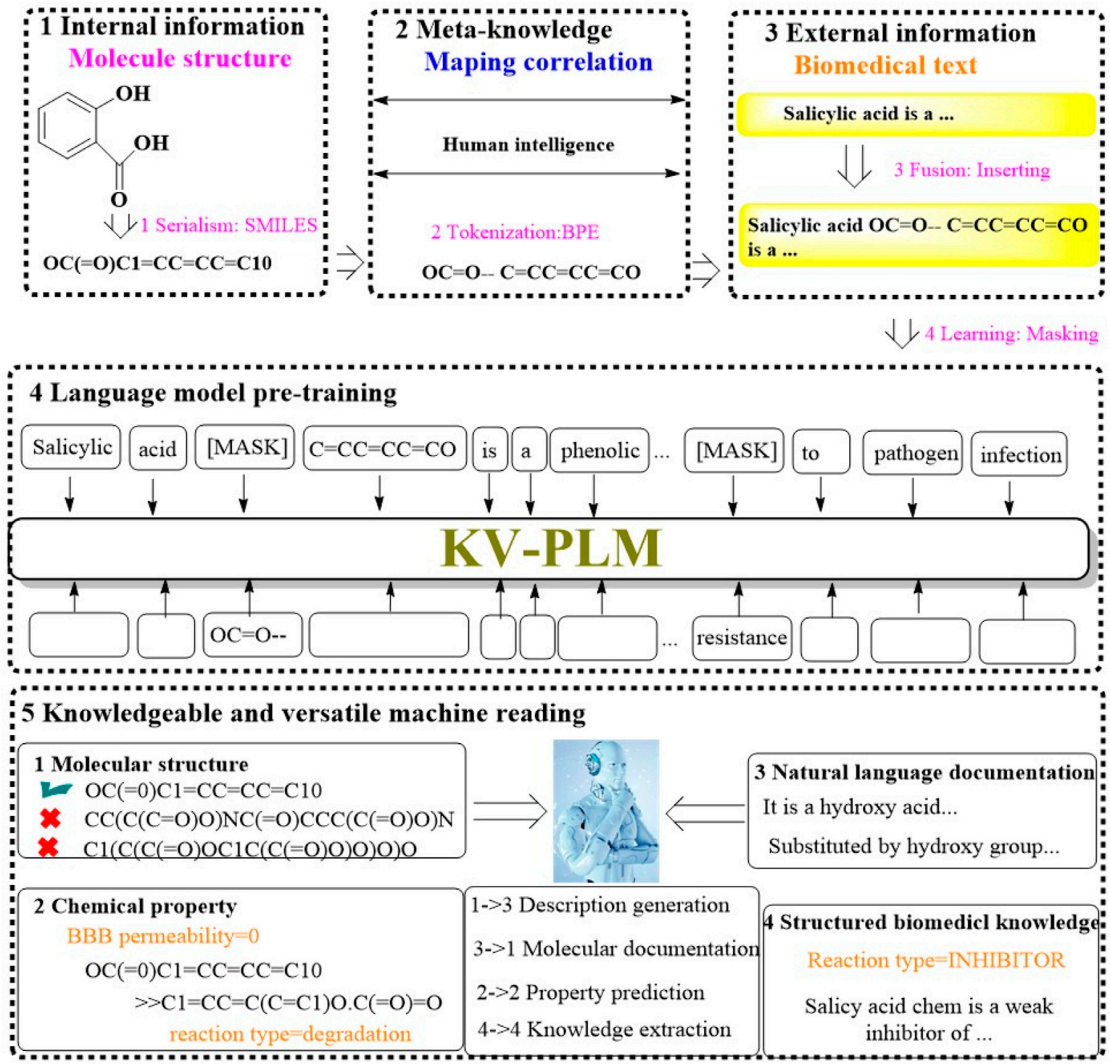
The architectural structure of KV-PLM. (The workflow of KV-PLM starts with internal molecular structure serialization into SMILES format, then the Meta-knowledge mapping is utilized to correlates molecular features to human intelligence. External biomedical text would subsequently be fused with molecular data. The language model pre-training would empower the model with the ability to predict molecular properties. It enables knowledgeable machine reading across different molecular properties (e.g., molecular structure, chemical properties, natural language documentation, and structured biomedical knowledge), which would facilitate tasks like description generation, property prediction, and knowledge extraction).

Recognizing the limitations of current machine reading models, which tend to handle various data types separately, a significant divide often emerges between the nuanced interpretation of molecular structures and the absorption of knowledge from biomedical literature. To address this, Recently, a groundbreaking machine reading system known as KV-PLM has been introduced. It is designed to seamlessly integrate molecular structure data with biomedical text within a single deep learning architecture (Figure 9) (Zeng et al., 2022). The KV-PLM leverages the pretrained language model BERT12 as its foundational component. It employs the simplified molecular-input line-entry system (SMILES) to encode molecular structures into a format compatible with the byte pair encoding (BPE) algorithm. This encoding process transforms SMILES strings into a series of substring patterns. These patterns are then integrated into a comprehensive biomedical data and subjected to pretraining under a unified language modeling framework. The culmination of this process is the acquisition of meta-knowledge through a self-supervised language model, which can be efficiently adapted through fine-tuning for specific application within the biomedical domain.

To evaluate the efficacy of KV-PLM, a range of mono-source biomedical tasks were conducted, encompassing both molecular structure-related and biomedical text-related tasks. The system’s performance was benchmarked on MoleculeNet for SMILES property classification across datasets such as BBBP, SIDER, TOX21, and HIV, as well as for chemical reaction classification was evaluated on USPTO 1k TPL dataset. Additionally, the system was evaluated on biomedical named entity recognition (NER) and the relation extraction (RE) task using BC5CDR and ChemProt. The results indicated that KV-PLM model not only outperformed other model in these tasks but also demonstrated an ability to engage in knowledgeable and versatile reading.

Moreover, the system’s proficiency in versatile reading tasks involving “cross-information retrieval”, “match judging”, and “human professional performance” was confirmed. KV-PLM excelled in these versatile tasks, notably in facilitating effective cross retrieval between substances and property descriptions. These capabilities highlight the immense potential of KV-PLM in the realms of novel drug discovery and molecular property prediction, offering researchers a tool to gain a holistic and in-depth comprehension of molecular entities.

#### 2.1.10 MolT5

**FIGURE 10:**
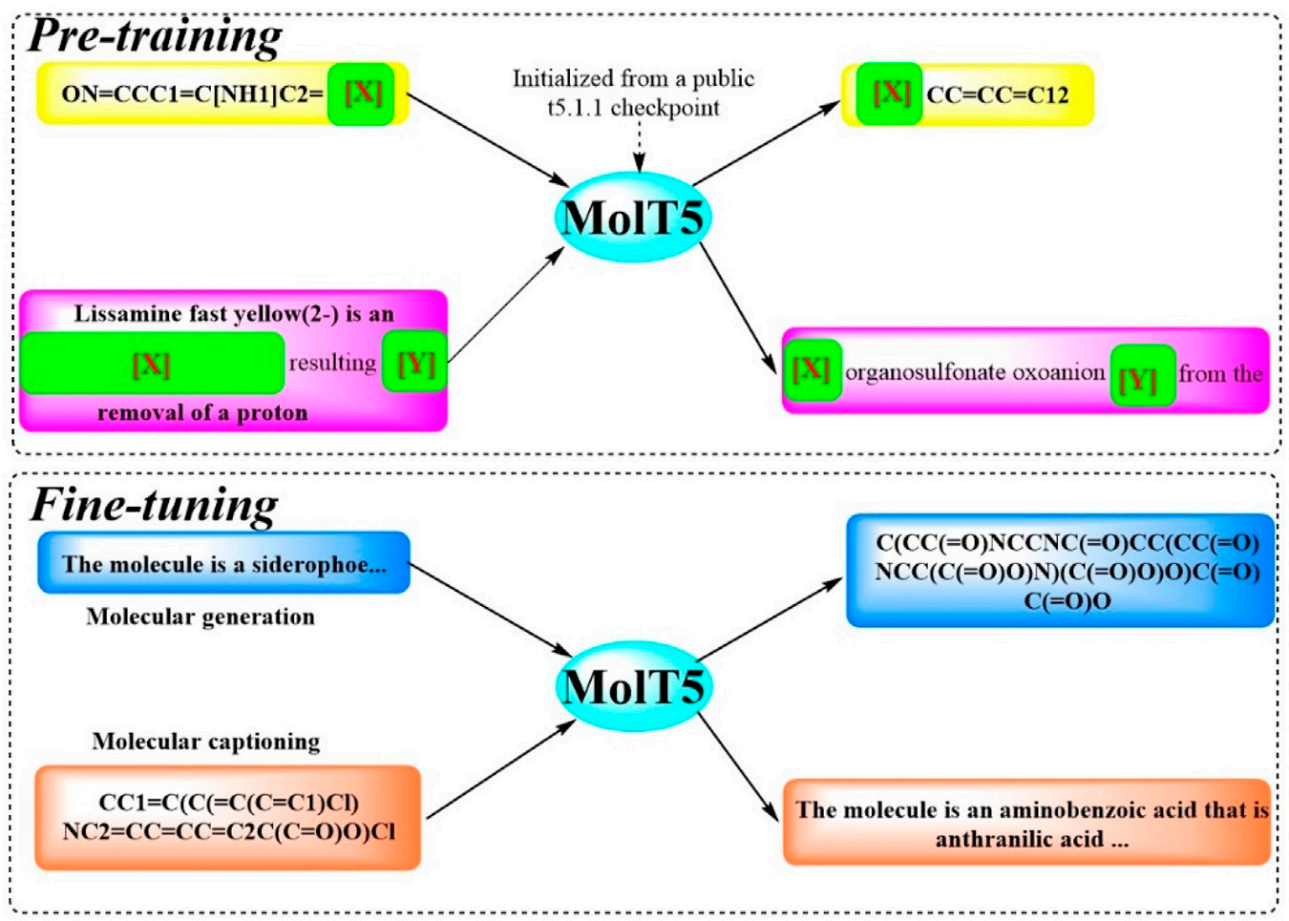
The workflow of MolT5. (MolT5 is trained in two steps (Sadybekov and Katritch, 2023): in pre-training, it learns from public data such as the chemical formulas and reactions (Murray et al., 2023). In fine-tuning, it specializes in tasks like creating molecule descriptions and generating new molecules).

To facilitate efficient communication between molecules structures and natural language, and to address challenge of limited data, Molecular T5 (MolT5), a self-supervised learning framework, was developed (Edwards et al., 2022). This framework employs a transformer-based architecture (Figure 10), leveraging the T5 that has been pretrained text-to-text model, enabling the simultaneous processing of vast amounts of unlabeled natural language text and molecular string data for pre-training purposes. In pursuit of this objective, two new tasks were introduced and formalized: molecular language tasks, specifically molecule captioning, and text-guided *de novo* molecule generation. Although these molecule–language tasks share similarities with vision–language tasks, there present distinct challenges, particularly the increased complexity of molecular captioning due to the diverse range of possible languages used for captioning. The performance of MolT5 was rigorously assessed using a suite of evaluation metrics, including BLEU, ROUGE, and METEOR, as well as a newly developed cross-modal retrieval similarity metric, the Text2Mol metric. When evaluated against the ChEBI-20 dataset using both the Text2Mol metric and BLEU metric, MolT5 achieved superior scores and outperformed RNNs and transformers in the two newly defined tasks. Notably, the performance of MolT5 further improved as the language model size increased.

MolT5 pretrains models on single-modal data, effectively mitigating the issue of data scarcity within the chemical domain. Furthermore, a variety metrics were also adopted including a new cross-modal embedding-based metric, to evaluate the performance of molecule captioning and text-based molecule generation. Results show that MolT5-based models are capable of generating high-quality outputs, encompassing both molecules and captions, in numerous instances.

As previously mentioned, the effective deployment of MolT5 poised to bolster the application of molecular AI, empowering researchers to uncover potential drug candidates by engaging with AI through natural language interactions and acquiring target chemical structures with specific functional attributes rather than relying solely on their properties. However, it is imperative to pay close attention to the potential biases introduced by the training dataset, the SMILES strings utilized, and the authenticity of the compounds listed in ChEBI-20.

#### 2.1.11 Text + Chem T5

**FIGURE 11:**
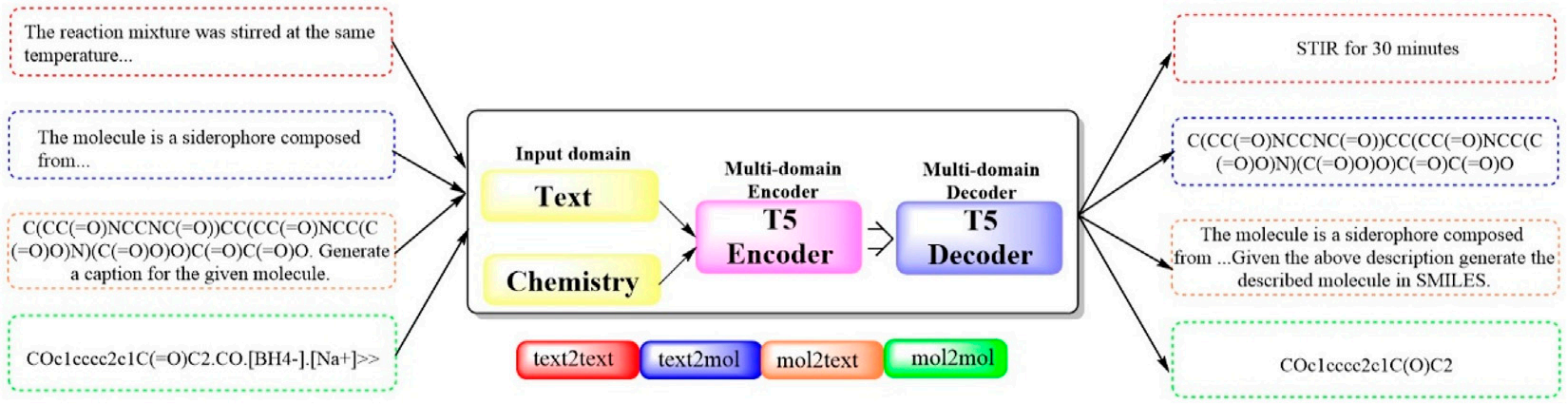
The pipeline of Text + Chem T5. (Text + Chem T5 is capable of dealing with both text and chemistry information, which has a text encoder and a chemistry encoder that work together to understand inputs from different domains. The model is capable of understanding a chemical structure and generate a description for it, or it could receive a description and generate the chemical structure in SMILES format).

To bridge the gap between human‒machine interactions and establish a cohesive framework for natural language and chemical representations, the first multidomain, multitask language model, Multitask Text and Chemistry T5 (Text + Chem T5 (Figure 11), https://github.com/GT4SD/gt4sd-core), was developed (Christofidellis et al., 2023). This model excels in managing both chemical and natural languages in parallel, outperforming others in cross-domain tasks across a broad spectrum of NLP-based evaluation metrics. Moreover, it negates the need for costly mono-domain pretraining and task-specific models. The capabilities of Text + Chem T5 was rigorously assessed across a range of tasks, including the predictions of forward and reverse chemical reactions, the generation of text-conditional novel molecules, the captioning of molecules spanning various domains, and execution of paragraph-to-action tasks within the linguistic domain. The findings underscored the effectiveness of Text + Chem T5 as a versatile multidomain and multitask model, adept at generating precise and enlightening captions (with a BLEU-2 score of 0.625, a Rouge-1 score of 0.647 and a Rouge-2 score of 0.498) and adeptly translating between natural language and the SMILES representation of molecules in both text-to-chemistry and chemistry-to-text endeavors.

In particular, Text + Chem distinguishes itself by its capacity to navigate complex drug discovery workflows, such as a hypothetical molecular discovery process, with a unified model. In this study, Text + Chem T5 uniquely succeeded in generating the desired molecule for the “text-to-SMILES task”, provide a synthetic route identical to the target reaction for “retrosynthesis”, and conceptually succeed in identifying and proposing an extremely similar reaction in a chemistry laboratory for a “paragraphs to actions” task. This fascinating capability, previously uncharted, positions Text + Chem T5 as superior even to established models such as ChatGPT and Galactica 1.3B.

The paramount advantage of Text + Chem T5 lies in its multifaceted task management. As indicated in this manuscript, Text + Chem is poised for targeted application across a variety of fields, such as chemical reaction prediction and retrosynthesis, significantly and efficiently bolstering modern drug development discovery in the physical sciences. It streamlines these processes by circumventing the need for task-specific fine-tuning and enhancing human–model interactions.

#### 2.1.12 Mol-Instructions

**FIGURE 12:**
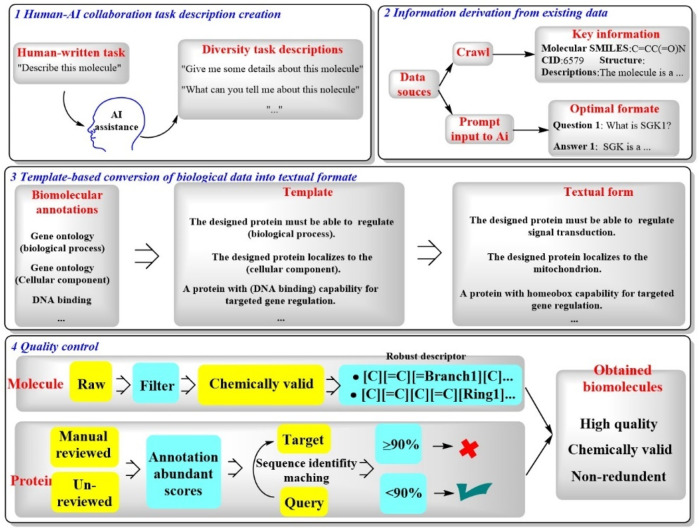
A four-step process of Mol-Instructions for creating and refining molecular descriptions and data. [(Sadybekov and Katritch, 2023) human and AI work together to generate task descriptions about molecules (Murray et al., 2023); information about molecules is gathered from data sources and formatted for AI (Cova et al., 2022); biological data is turned into text using templates, such as description of the functions of a specific protein (Roggia et al., 2024); the quality of molecules and proteins is checked].

Recently, a novel LLM named Mol-Instructions has been introduced, specifically crafted to address the complexities of biomacromolecules, particularly those relevant to structural biology (Fang et al., 2023a). This model encompasses a comprehensive instruction dataset that is segmented into three pivotal components (Figure 12). (Sadybekov and Katritch, 2023) Molecule-oriented instructions, which delve into the inherent properties and behaviors of small molecules essential for chemical reactions and molecular design (Murray et al., 2023); protein-oriented instructions, which are geared towards predicting the structures, functions, and activities of proteins for protein design; and (Cova et al., 2022) biomolecular text instructions, which engage in natural language processing (NLP) tasks that are integral to the fields of associated with bioinformatics and cheminformatics.

As shown in Figure 12, Mol-Instructions demonstrates great potential in biomolecular studies. In particular, Mol-Instructions could be applied in three major areas (Sadybekov and Katritch, 2023): assessment of cross-modal comprehension, which involves the integration of different types of data to enhance understanding of biomolecular systems (Murray et al., 2023). Exploration of deeper biomolecular design, enabling the development of more sophisticated and effective molecular structures (Cova et al., 2022). Tool learning to address complex biological challenges, leveraging advanced computational methods to address intricate biological questions. Mol-Instructions stands as a significant advancement in the integration of computational linguistics and molecular biology, offering a multifaceted approach to understanding and manipulating biomacromolecules.

#### 2.1.13 ConPLex (https://ConPLex.csail.mit.edu)

**FIGURE 13:**
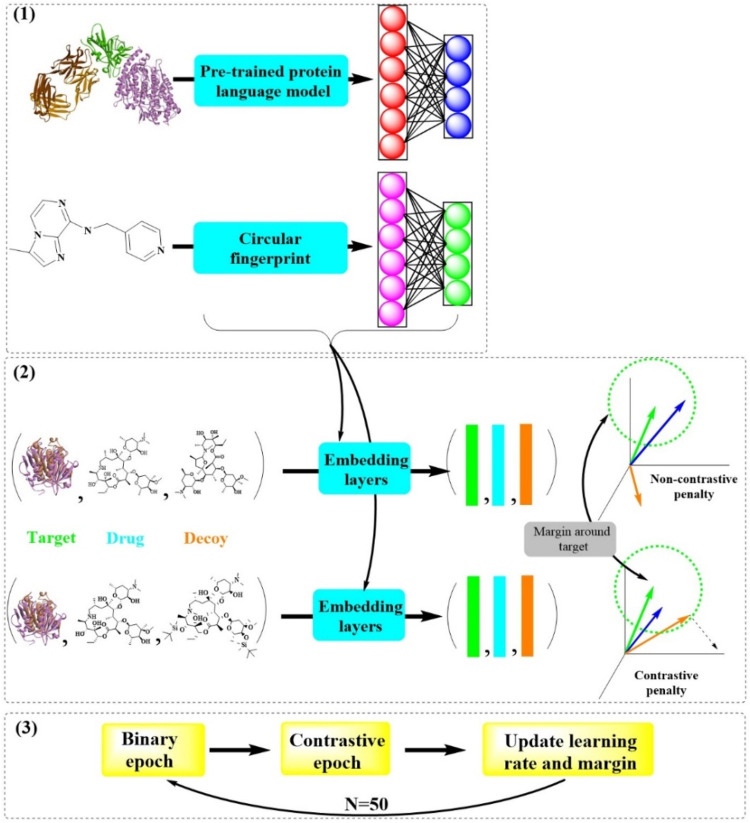
Outline of the ConPLex model architecture and training framework (In step (Sadybekov and Katritch, 2023), a pre-trained protein language model and a circular fingerprint method are used to analyze molecular structures. In step (Murray et al., 2023), embedding layers process the molecular data, creating a numerical representation. In step (Cova et al., 2022), the model undergoes a series of binary and contrastive epochs to update and refine the learning rate and margin for improved accuracy in predicting molecular interactions).

Recently, a sophisticated deep learning model known as ConPLex has been successfully developed for the sequence-based prediction of drug-target interactions with remarkable accuracy, broad adaptivity, and specificity (Singh et al., 2023). ConPLex boasts a competitive edge due to the innovative integration of pretrained protein language models (“PLex”, for lexicographic pretraining) and protein-anchored contrastive coembedding (“Con”, for contrastive learning) (Figure 13). The “PLex” component is capable of alleviating challenges posed by limited DTI training data, while the “Con” aspect effectively maps target proteins and drugs into a unified latent space, ensuring distinct separation between true interacting partners. Consequently, ConPLex enables more accurate predictions of DTIs by leveraging the distance within the learned representations, even when dealing with massive compound libraries and the expansive human proteome.

The experimental results have demonstrated the model’s efficacy in successfully predicting the tested kinase-drug interactions, with 12 out of 19 pairs showing *K*_*D*_ values less than 100 nM, including four with subnanomolar affinity and an efficient EPHB1 inhibitor (PD-166326, *K*_*D*_ = 1.3 nM). Beyond its broad generalizability and high specificity, ConPLex enhances interpretability, rendering the drug-target embedding space and the functions of human cell-surface proteins more transparent. In addition to the *in silico* screening of small-molecular-weight compounds, ConPLex holds potential for screening other drugs types, such as antibodies, and for toxicity prediction.

Given these significant advantages, ConPLex is anticipated to revolutionize *in silico* drug screening at the genomic scale and to accelerate the development of innovative drugs in modern pharmaceutical research.

### 2.2 Deep learning for macromolecular drugs (protein structure prediction)

Protein structures are traditionally elucidated through experimental techniques such as by X-ray crystallography, nuclear magnetic resonance (NMR) and electron cryomicroscopy (cryo-EM), which are known for their precision. However, these methods are complex, time-consuming, and costly, which limits their widespread application. In light of these constraints and the growing need for novel protein structures, there has been a surge in interest in innovative strategies, particularly bioinformatics approaches to obtain novel protein structures. Despite the promise of these methods, they still necessitate considerable experimental effort.

#### 2.2.1 AlphaFold

AlphaFold, developed by DeepMind, has revolutionized protein structure prediction with unprecedented accuracy and reliability, harnessing the power of neural networks and homology modeling for protein model construction (Pandey et al., 2022). To extend the capabilities to predict protein complexes accurately and efficiently, AlphaFold-Multimer was introduced, expanding the capabilities of Alphafold2 to handle multiple chains (Yin et al., 2022). The latest version, AlphaFold3, has been successfully applied in various fields, including modeling of conventional protein structures and structures with novel folds, structural construction of artificial constructs and prediction of protein‒protein interactions. In particular, models generated by AlphaFold typically achieve TM-scores greater above 0.9, suggesting that both the overall fold and the details of the constructed models are theoretically correct (Skolnick et al., 2021). To date, AlphaFold DB (AlphaFold DB, https://alphafold.ebi.ac.uk) has provided open access to more than 214 million protein structure predictions (Varadi et al., 2023).

The exceptional performance of AlphaFold is largely attributed to the novel neural network architectures and specialized training regimens that incorporate evolutionary, physical and geometric constraints inherent to protein structures (Jumper and Hassabis, 2022). Alongside the simultaneous generation of multiple sequence alignments (MSAs) and pairwise features, two key modules, Evoformer and the structure module, play critical roles in protein structure development (Skolnick et al., 2021). Evoformer, a building block of a novel neural network, approaches predict protein structures as a graph inference problem, with graph edge defined by the proximity of residues. It consists of two specialized transformers for distinct data types: the MSA transformer and the pair representation transformer. The structure module is tasked with local side chain packing rearrangements, prioritizing the orientations of the protein backbone and residues, and positioning the side chains of different residues.

In a study utilizing the program Accuracy of NMR Structures Using RCI and Rigidity (ANSURR), the accuracy of AlphaFold-generated structures was compared to NMR structures (Fowler and Williamson, 2022). The results revealed that the AlphaFold models generally surpass NMR ensembles in accuracy, although there are scenarios, particularly those involving dynamic structures, where NMR ensembles may be more precise. This suggests that AlphaFold might display relatively low confidence in predicting dynamic structures. Consequently, it has been proposed that AlphaFold could be instrumental in refining NMR structure. Furthermore, structures generated by AlphaFold and subsequently validated by ANSURR are likely to satisfy application requirements, potentially eliminating the need for additional refinement processes.

#### 2.2.2 MULTICOM

To enhance the precision of AlphaFold-Multimer in predicting complex structure (Zhu et al., 2023), a sophisticated quaternary structure prediction system (MULTICOM) has been developed (Liu et al., 2023b). It is capable of optimizing the inputs transformed into AlphaFold-Multimer, evaluating and refining the resulting outputs. It employs a dual approach, utilizing traditional sequence alignment and Foldseek-based structure alignment to generate MSAs and to identify templates for individual monomers. These MSAs for monomers are subsequently merged to form MSAs for multimers. Moreover, the structural predictions generated can be appraised using a suite of complementary metrics, and the refinement of structural predictions can be achieved through a Foldseek-based structure alignment strategy.

The results showed that the average TM-score for the initial predictions from MULTICOM for CASP15 assembly targets was ∼0.76, making a 5.3% increase over the standard AlphaFold-Multimer. The average TM-score for the top 5 predictions by MULTICOM was ∼0.80, which represents an 8% increase compared with the standard AlphaFold-Multimer. In addition, the Foldseek structure alignment-based multimer structure generation (FSAMG) method outperformed several prevalent sequences alignment-based multimer structure generation methods, such as NBIS-AF2-multimer predictions.

#### 2.2.3 ComplexQA

**FIGURE 14:**
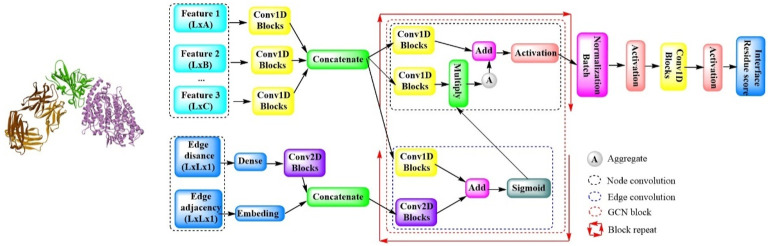
The model architecture of ComplexQA with the deep graph convolution network. (ComplexQA is capable of molecular analysis that processes various molecular features through Conv1D blocks and integrates edge information using Conv2D blocks. Then applies graph convolutional networks (GCNs) with multiple blocks was used for feature aggregation and normalization. Finally, it concludes with the generation of a residue interface score to assess molecular interactions).

In a cutting-edge study, a novel model quality assessment method, ComplexQA, has been introduced. This method leverages a deep graph neural network-based algorithm designed to assess the local quality of interfacial residues within protein complexes (Figure 14) (Zhang et al., 2023). It does so by analyzing a combination of sequence data, 3D structural information, and chemical properties. The process begins by converting the protein complex structures into undirected graphs, followed by the derivation of feature representation for each graph node. All the features, including the hidden features, are concatenated and used for graph learning purposes. To represent the edges of the graph, residue–residue features are acquired, primarily through two newly designed matrices: the adjacent matrix and the edge distance matrix. By integrating these two representations, the edge embedding features are generated, which are then employed for the subsequent edge convolution operations within the graph convolutional network block. This block further consists of two subblocks: one for edge convolution and another for node convolution. Finally, the output is transformed into a 1D convolutional layer, which employs a linear activation function to produce the final results. This sophisticated approach by ComplexQA offers a comprehensive evaluation of protein complex structures, enhancing our ability to understand and predict their quality.

In comparative evaluations across diverse datasets, ComplexQA outperformed the other leading algorithms (DProQA, GNN-DOVE, TRScore, GOAP, and ZRANK2). It also displayed commendable performance on challenging targets that featured a sparse number of acceptable models. Furthermore, ComplexQA is capable of delivering a detailed assessment of each interface residue, offering a level of precision that is invaluable in the field of protein complex structure analysis.

#### 2.2.4 ProtGPT2

ProtGPT2 (https://huggingface.co/nferruz/ProtGPT2), a cutting-edge autoregressive transformer model grounded in language-based principles, has been engineered to *de novo* construct protein structures with high throughput efficiency (Ferruz et al., 2022). The transformer was trained on an expansive dataset of ∼50 million non-annotated sequences from the UniRef50 (UR50) database, encompassing the full spectrum of protein diversity, thereby enabling it to learn and “comprehend” the intricacies of protein language in an autoregressive manner. In addition to the standard performance metrics, a suite of extrinsic tests was meticulously designed to assess the quality of the protein sequences generated by ProtGPT2.

The findings were compelling that ProtGPT2 demonstrated an impressive ability to generate sequences that, while remotely related to natural counterparts, also bore resemblance to known structural spaces. The generated proteins mirrored the natural amino acid propensities observed in their naturally occurring counterparts, with a notable predilection for globular structures, accounting for roughly 80% of the generated proteins. Moreover, sequences generated by ProtGPT2 were found to be only distantly related to those found in nature. When these results were integrated with similarity network analyses, it became evident that ProtGPT2 possesses the unique capability to explore and sample previously uncharted territories within the vast protein space.

#### 2.2.5 ProteinMPNN

**FIGURE 15:**
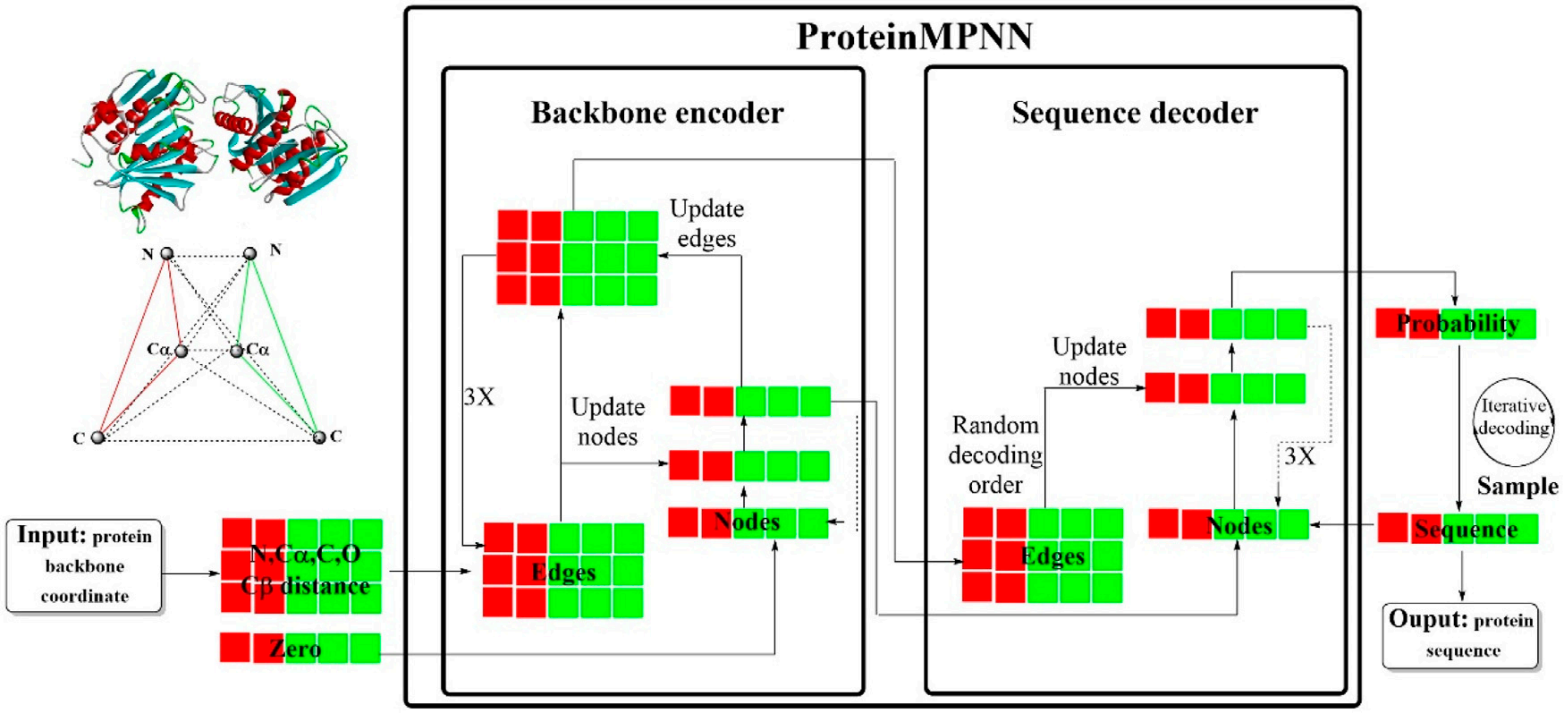
The workflow of ProteinMPNN. (ProteinMPNN is consisted of a backbone encoder that processes protein backbone coordinates and edge information, and a sequence decoder capable of generating protein sequences. Nodes and edges are updated in the encoder, and through iterative decoding, it produces a protein sequence with a calculated probability).

Recently, a novel deep learning-based protein sequence design strategy, ProteinMPNN, has emerged, demonstrating significant advantages in both *in silico* and experimental tests (Dauparas et al., 2022). This innovative approach is founded on the structured transformer framework (Figure 15), incorporating a message-passing neural network (MPNN) architecture that encompasses 3 encoder layers, 3 decoder layers and 128 hidden layers. ProteinMPNN was designed to predict target protein sequences in an autoregressive manner from the N- to C-terminus using protein backbone features as input data. The sequence recovery rate of the baseline model was approximately 41.2%, which was notably increased to more than 50% following a series of improvements. These enhancements were primarily focused on the following aspects (Sadybekov and Katritch, 2023): incorporating additional distance metrics between virtual Cβs (Murray et al., 2023), introducing an edge update mechanism (Cova et al., 2022), employing random sampling for the decoding order within the decoder (Roggia et al., 2024), integrating coding information regarding the relative position and chain number, and (Pandey et al., 2022) the incorporation of Gaussian noise to enhance model robustness.

The results were impressive, with ProteinMPNN demonstrating the ability to design sequences for monomers and cyclic oligomers with remarkable stability and precision. Most of the proteins produced by the MPNN were soluble (96 design sequences, 73 soluble). The crystal structures and the electron cloud density of the core side chain were highly consistent with the intended design structures. Similar successes were achieved in the design of proteins with cyclic and internal repeat symmetries as well as those incorporating polyproline II helix motifs.

ProteinMPNN achieved a sequence recovery rate of 52.4%, marking a 19.5% increase compared to that of Rosetta for native protein backbones, and even surpassed AlphaFold in this specific task. The running time was remarkably swift, averaging approximately one second. In particular, ProteinMPNN also enabled the coupling of amino acid sequences at various positions across single or multiple chains, further expanding its versatility and applicability in protein sequence design.

## 3 Concluding remarks

AI has become an indispensable tool for addressing a multitude of societal challenges. The future of AI in drug development is set to be a landscape of innovation and efficiency, and it has been seeing a significant shift towards data-driven approaches, personalized medicine and clinical trials revolution. Take the AI-driven drug discovery and personalized medicines for examples. AI is expected to dominate drug discovery by making more accurate predictions of drug-target interactions and enhancing our understanding of disease physiopathology. AI models will be trained on larger biomedical datasets, including genomics, proteomics, and metabolomics, to identify novel drug candidates and optimize drug design. AI will continue to drive the growth of personalized medicines by leveraging Big Data to tailor treatments to individual patients. The ability to analyze genetic, environmental, and lifestyle data will lead to the development of highly personalized treatment plans. AI has the potential to revolutionize clinical trials by improving patient recruitment, monitoring, and data analysis. Advanced algorithms will enable the identification of suitable candidates based on genetic and phenotypic profiles, ensuring that trials are conducted with the most appropriate cohort of participants. Particularly noteworthy is the proliferation of AI algorithm programs, including DeepMind AlphaFold, Atomwise, Recursion Pharmaceuticals, BenevolentAI, and Insilico Medicine. These examples showcase the diverse integration of AI across the drug development spectrum, from the early stages of drug discovery to manufacturing processes and post-market surveillance. The future looks promising, with AI set to play a central role in making drug development more efficient, targeted, and personalized.

Nevertheless, the proliferation of LLMs ha as also sparked significant apprehensions, such as the phenomenon of “artificial hallucinations” (Beutel et al., 2023; Ji et al., 2022). The dissemination of AI-generated misinformation, fiction, or unsubstantiated claims poses a risk of misguiding unsuspecting users. To optimize benefits and mitigate risks, several key challenges must be surmounted to harness the full potential of LLMs (Sadybekov and Katritch, 2023). Transparency concerns. This is paramount for academic discourse surrounding generative AI. It is recommended that the judicious use of AI in scientific research be underscored and clearly articulated, as this could significantly bolster credibility (Tang et al., 2024). Therefore, tools and techniques that enhance the explain ability and interpretability of AI models are crucial. Moreover, the transparency in data governance is essential, providing insight into the quality and suitability of data used for training and inference in algorithmic decision-making. This includes documenting the origin of data, collection methods, and any preprocessing steps, which is crucial for identifying and mitigating potential biases (Murray et al., 2023). Combating AI hallucinations. AI hallucinations can occur due to several factors, including overfitting, training data bias/inaccuracy, and high model complexity. The foundation of preventing AI hallucinations lies in using high-quality, diverse training data that represents real-life scenarios without biases and errors. Moreover, regular validation using test datasets and human-in-the-Loop verification should also be instituted to preempt the spread of misinformation and to counteract biased responses. Finally, risk-based review systems and retrieval-augmented generation (RAG) might also be helpful in prioritizing and verifying the review of AI outputs (Cova et al., 2022). Dataset limitations. Owing to the nature of AI, the quality and scope of available data are pivotal to the design and practical application of AI models. There is a pressing need to focus on the quantity and quality of data, with larger and more diverse datasets being crucial for enhancing model performance (Roggia et al., 2024). Building trust in models. Trust is established through a combination of technical reliability, transparency, and alignment with user expectations. The factors that foster trust in models predominantly center on selecting the appropriate neural network architecture and molecular representations, alongside the advancement of innovative architectures imbued with inductive bias. For instance, recurrent neural networks (RNNs) are well-suited for sequential data due to their ability to maintain a form of memory. However, the choice extends beyond RNNs to include other architectures like convolutional neural networks (CNNs), which are effective for image data, and graph neural networks (GNNs), which are particularly adept at handling graph-structured data like molecules. As for molecular representations, except for SMILES strings, graph representations could capture the molecular structure more directly, including both topological and geometrical information, which is essential for tasks like drug discovery and material science. Moreover, innovative architectures with inductive bias (e.g., the 3D-CNN architecture (Skalic et al., 2019)) should be further developed to better address the nuances of specialized tasks. For example, geodesic 3D convolutional neural networks (gCNNs) use geodesic convolutions that consider the intrinsic geometry of the data, which is particularly useful in medical applications where the curvature and shape of organs, bones, and tissues are critical. These architectures can lead to improved model accuracy and computational efficiency by focusing on the most important information in the data (Pandey et al., 2022). Data safety and privacy. The safeguarding of personal information in terms of security, privacy, and confidentiality is non-negotiable, especially in the context of research, standards development, and commercial applications (Jayatunga et al., 2022). Computational complexity (time complexity, space complexity and scalability). The challenges posed by the computational demands and intricacies of contemporary deep learning methods are expected to remain a significant factor in the near term. For example, to address the scalability challenge, many AI applications leverage distributed computing and parallel processing techniques. Reducing the computational complexity of deep learning models can be achieved through network compression and acceleration techniques. Moreover, quantum computing offers a potential solution to overcoming computational limitations in AI. Quantum algorithms for machine learning, such as Grover’s algorithm, can potentially reduce the complexity of certain tasks, making previously intractable problems solvable. Additionally, quantum neural networks leveraging qubits could operate with higher efficiency and improved processing speed.

With unwavering conviction, we are stand atop the pinnacle of research, an epoch where AI, and especially LLMs, are set to transcend mere advancement and emerge as vital pillars of contemporary pharmaceutical innovation. It is imperative to accentuate the sophisticated and proficient application of AI throughout the biotechnological pharmaceutical development continuum, demonstrating its unparalleled ability to catalyze scientific breakthroughs and augment the efficacy of drug discovery endeavors.
